# Supplementary material for: Description of Three Novel Members in the Family Geobacteraceae, Oryzomonas japonicum gen. nov., sp. nov., Oryzomonas sagensis sp. nov., and Oryzomonas ruber sp. nov
Source: Microorganisms. 2020 Apr 27;8(5):634. doi: 10.3390/microorganisms8050634 (PMC7285026; doi:10.3390/microorganisms8050634)
Supplement: Supplementary file 1 [file microorganisms-08-00634-s001.pdf]

microorganisms

Supplementary Material

Description of three novel members in the family *Geobacteraceae*, *Oryzomonas japonicum* gen. nov., sp. nov., *Oryzomonas sagensis* sp. nov., and *Oryzomonas ruber* sp. nov.

Zhenxing Xu [1], Yoko Masuda [1*], Chie Hayakawa [2], Natsumi Ushijima [3], Keisuke Kawano [4†], Yutaka Shiratori [5], Keishi Senoo [1, 6], Hideomi Itoh [7*]

[1]Department of Applied Biological Chemistry, Graduate School of Agricultural and Life Sciences, The University of Tokyo, Tokyo 113-8657, Japan

[2]School of Agriculture, Utsunomiya University, Tochigi 321-8505, Japan

[3]Support Section for Education and Research, Graduate School of Dental Medicine, Hokkaido University, Hokkaido 060-8586, Japan

[4]Department of Marine Biology and Sciences, School of Biological Sciences, Tokai University, Hokkaido 005-8601, Japan

[5]Niigata Agricultural Research Institute, Niigata 940-0826, Japan

[6]Collaborative Research Institute for Innovative Microbiology, The University of Tokyo, Tokyo 113-8657, Japan

[7]Bioproduction Research Institute, National Institute of Advanced Industrial Science and Technology (AIST) Hokkaido, Hokkaido 062-8517, Japan

[†]Present address: Division of Agriculture, Graduate School of Agriculture, Hokkaido University, Hokkaido 060–8589, Japan

Author for correspondence:

Dr. Yoko Masuda

Email: ygigico@gmail.com

Dr. Hideomi Itoh

Email: hideomi-itou@aist.go.jp

Supplementary Tables

Table S1. The 16S rRNA gene similarities (%) between the three novel strains and other type strains in the family *Geobacteraceae.*

Reference strains	Accession numbers	16S rRNA gene similarity (%)		
		**Red96^T**	**Red100^T**	**Red88^T**
Oryzomonas japonicum Red96^T	MK334373	100		
Oryzomonas sagensis Red100^T	MK334374	99.44	100	
Oryzomonas ruber Red88^T	MK334372	99.79	99.37	100
Pelobacter propionicus DSM 2379^T	CP000482	95.66	95.38	95.73
Geobacter lovleyi SZ^T	CP001089	95.66	95.45	95.73
Geobacter chapellei 172^T	U41561	95.64	95.36	95.71
Geobacter thiogenes ATCC BAA-34^T	FUWR00000000	95.59	95.52	95.66
Geobacter psychrophilus P35^T	NR_043075	94.66	94.38	94.73
Geobacter luticola OSK6^T	AB682759	94.60	94.46	94.67
Geobacter metallireducens GS-15^T	CP000148	92.36	92.22	92.43
Geobacter uraniireducens Rf4^T	CP000698	94.10	93.82	94.03
Geobacter toluenoxydans JCM 15764^T	BBCJ00000000	94.73	94.44	94.66
Geobacter daltonii FRC-32^T	CP001390	94.59	94.31	94.52
Geobacter grbiciae TACP-2^T	AF335182	92.43	92.29	92.50
Geobacter hydrogenophilus H2^T	U28173	92.44	92.29	92.44
Geobacter sulfurreducens PCA^T	AE017180	92.22	92.01	92.22
Geobacter anodireducens SD-1^T	CP014963	92.07	92.07	92.00
Geobacter pickeringii G13^T	CP009788	92.84	92.77	93.12
Geobacter soli GSS01^T	JXBL00000000	92.08	92.08	92.01
Geobacter argillaceus ATCC BAA-1139^T	VLLN00000000	93.26	92.91	93.19
Geobacter pelophilus Dfr2^T	U96918	93.68	93.40	93.75
Geomonas oryzae S43^T	MH915553	93.75	93.54	93.68
Geomonas edaphica Red53^T	MH915554	93.75	93.54	93.68
Geomonas ferrireducens S62^T	MH915555	93.61	93.40	93.54
Geomonas terrae Red111^T	MH915556	93.47	93.26	93.40
Geomonas bemidjiensis Bem^T	CP001124	93.89	94.03	93.82
Geomonas bremensis Dfr1^T	U96917	93.53	93.53	93.46

Table S2. Annotated genes involved in ferric reduction of the three novel species

Gene symbol	Annotation	Accession numbers in NCBI database		
		O. japonicum **Red96^T**	***O. sagensis*** **Red100^T**	***O. ruber*** **Red88^T**
omcB	lipoprotein cytochrome *c*	WP_151128903.1	WP_151154925.1	WP_149307804.1
omcC	lipoprotein cytochrome *c*	WP_151127861.1	WP_151156234.1	WP_149306618.1
ombB/ombC	OM integral protein	WP_151126229.1	WP_151155031.1	WP_149310033.1
omaB/omaC	cytochrome *c*	WP_151129059.1	WP_151155030.1	WP_149305526.1
ppcA	periplasmic cytochrome *c*	WP_151128488.1	WP_151156562.1	WP_149306251.1
cbcL	menaquinol oxidoreductase complex Cbc7	WP_151126363.1	WP_151155206.1	WP_149307569.1

Table S3. Annotated genes involved in pilin synthesis of the three novel species

Gene symbol	Annotation	Seed ortholog#	Accession numbers in NCBI database		
			***O. japonicum* Red96^T**	***O. sagensis* Red100^T**	***O. ruber* Red88^T**
pilA	prepilin-type N-terminal cleavage/methylation domain-containing protein	243231.GSU1496	WP_151127625.1.	WP_151157262.1	WP_149305846.1
pilT-3	Type II/IV secretion system protein	338966.Ppro_0536	WP_149307554.1	WP_151155222.1	WP_149307554.1
pilT-1	PFAM type II secretion system protein E	338966.Ppro_3178	WP_151126839.1	WP_151155672.1	WP_149307317.1
pilC	PFAM type II secretion system	338966.Ppro_2512	WP_151127599.1	WP_151157291.1	WP_149305818.1
pilT-4	Type II/IV secretion system protein	338966.Ppro_2513	WP_151127600.1	WP_151157290.1	WP_149305819.1
pilB	General secretory system II, protein E domain protein	338966.Ppro_2514	WP_151127601.1	WP_151157289.1	WP_149305820.1
pilH	PFAM ABC transporter related	338966.Ppro_1661	WP_151127620.1	WP_151157267.1	WP_149305841.1
pilI	ABC-type transport system involved in multi-copper enzyme maturation permease component	338966.Ppro_1659	WP_151127622.1	WP_151157265.1	WP_149305843.1
pilR	Response regulator	338966.Ppro_1655	WP_151127626.1	WP_151157261.1	WP_149305847.1
pilM	TIGRFAM type IV pilus assembly protein PilM	338966.Ppro_0992	WP_151128702.1	WP_151157065.1	WP_149309234.1
pilN	Fimbrial assembly protein (PilN)	338966.Ppro_0993	WP_151128703.1	WP_151157066.1	WP_149309232.1
pilO	Pilus assembly protein, PilO	338966.Ppro_0994	WP_151128704.1	WP_151157067.1	WP_149309230.1
pilP	Pilus assembly protein, PilP	338966.Ppro_0995	WP_151128705.1	WP_151157068.1	WP_149309228.1
pilQ	PFAM type II and III secretion system protein	338966.Ppro_0996	WP_151128706.1	WP_151157069.1	WP_149309227.1
pilD	Cleaves type-4 fimbrial leader sequence and methylates the N-terminal (generally Phe) residue	338966.Ppro_0982	WP_151128900.1	WP_151157542.1	WP_149305913.1
pilV-2	Pfam: N_methyl_2	338966.Ppro_0989	WP_151128849.1	WP_151157209.1	WP_149305906.1
pilG	Tetratricopeptide repeat	404380.Gbem_2587	WP_151127621.1	WP_151157266.1	WP_149305842.1

The codes of seed ortholog were used to fetch fine-grained orthologs and confirm related genes for the three novel species.

Table S4. The assembly accession numbers and genome size of the reference strains for genomic comparison. All the sequences were retrieved from NCBI database.

Reference strains	Accession numbers	Genome size (Mbp)
Oryzomonas japonicum Red96^T	VZQZ00000000	3.6
Oryzomonas sagensis Red100^T	VZRA00000000	3.6
Oryzomonas ruber Red88^T	SRSD00000000	3.8
Pelobacter propionicus DSM 2379^T	NC_008609.1	4.2
Geobacter lovleyi SZ^T	NC_010814.1	4.0
Geobacter thiogenes ATCC BAA-34^T	FUWR01000042.1	3.6
Geobacter metallireducens GS-15^T	NC_007517.1	4.0
Geobacter uraniireducens Rf4^T	NC_009483.1	5.1
Geobacter toluenoxydans JCM 15764^T	BBCJ01000001.1	4.2
Geobacter daltonii FRC-32^T	NC_011979.1	4.3
Geobacter sulfurreducens PCA^T	NC_002939.5	3.8
Geobacter anodireducens SD-1^T	NZ_CP014963.1	3.7
Geobacter pickeringii G13^T	NZ_CP009788.1	3.6
Geobacter soli GSS01^T	NZ_CP009788.1	3.7
Geomonas oryzae S43^T	RAHW00000000	4.9
Geomonas edaphica Red53^T	SSYB00000000	4.8
Geomonas ferrireducens S62^T	SSYA00000000	4.8
Geomonas terrae Red111^T	SRSC00000000	4.7
Geomonas bemidjiensis Bem^T	NC_011146.1	4.6
Geomonas bremensis R1	AUGE01000001.1	4.7

Supplementary Figures

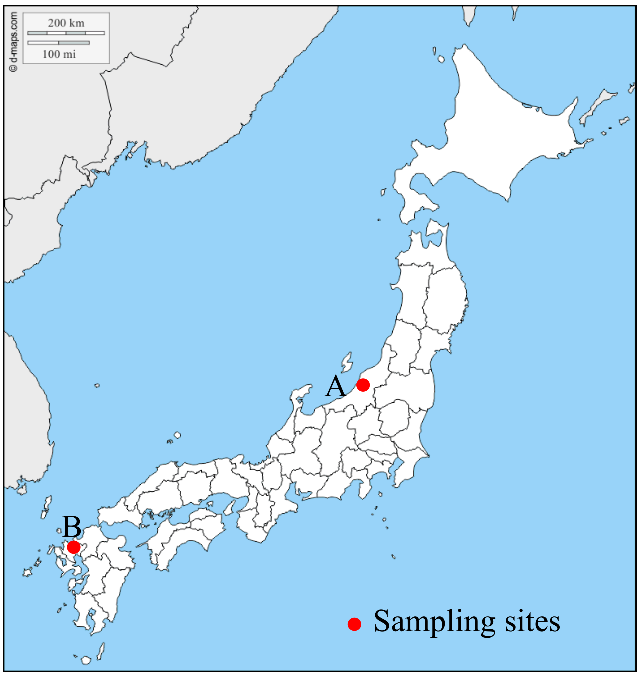

Figure S1. The geographical map of the sampling sites. A, Niigata, Japan, where strains Red96^{T} and Red88^{T} isolated; B, Saga, Japan, where strain Red100^{T} isolated.

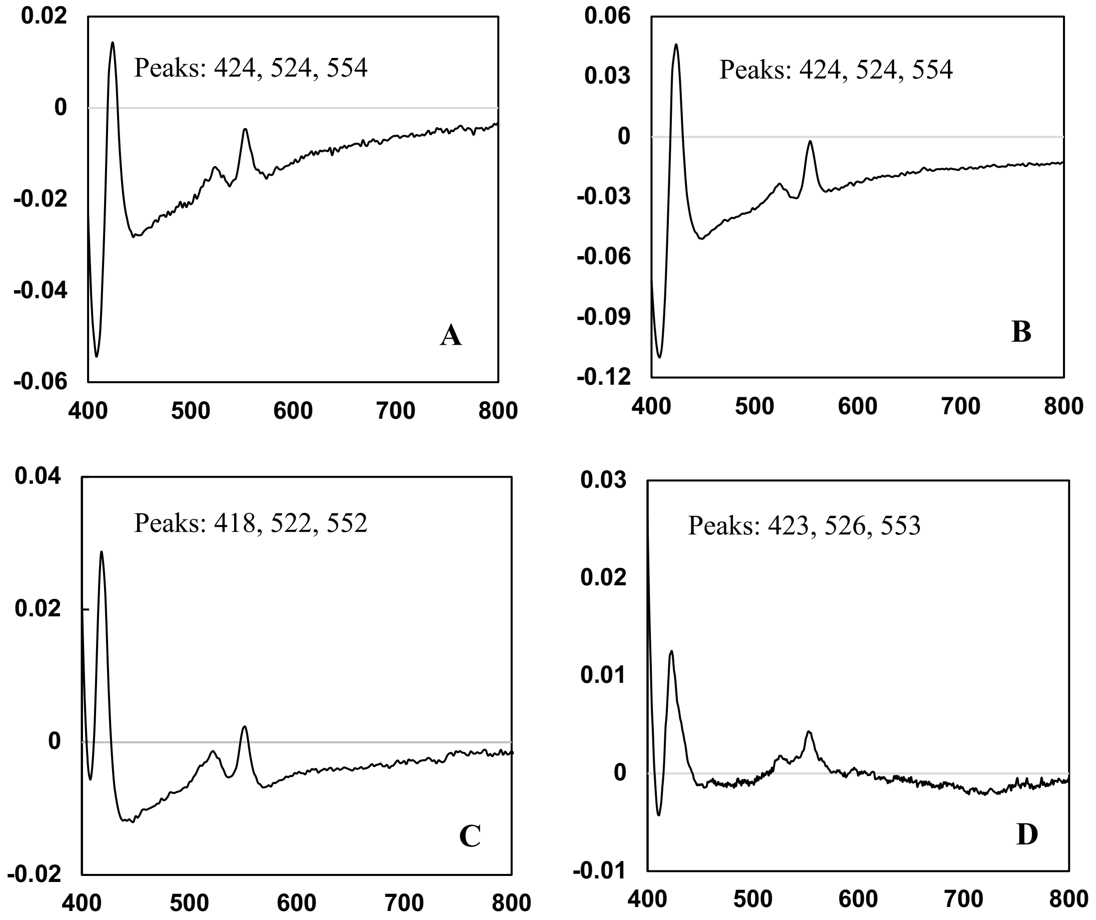

Figure S2. Difference spectrum of whole cells for the four analyzed strains in the wavelength range of 400–800 nm. *Oryzomonas japonicum* Red96^{T} (A), *Oryzomonas sagensis* Red100^{T} (B), *Oryzomonas ruber* R88^{T} (C) and *Geobacter chapellei* DSM 13688^{T} (D). The peak values shown in the figures were wavelength of every peak.

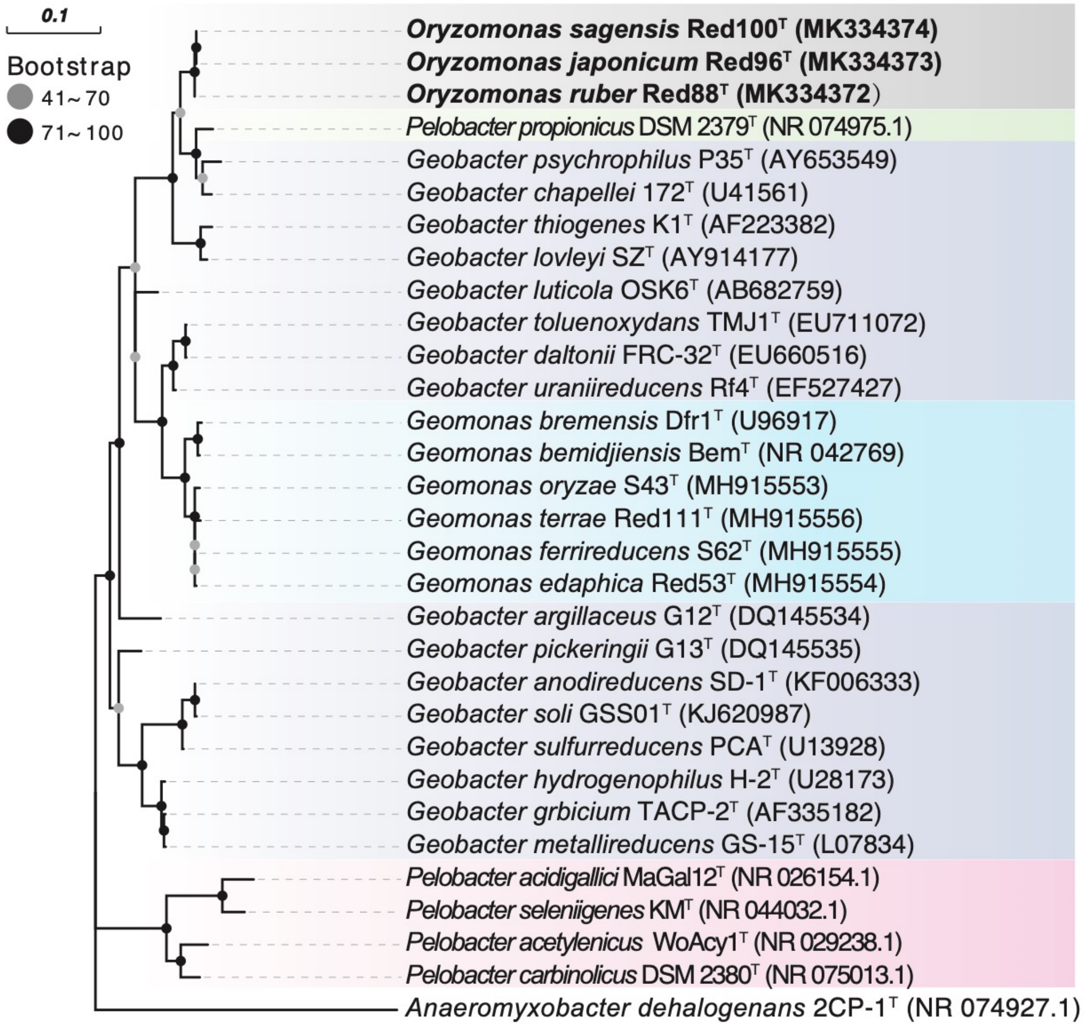

Figure S3. Phylogenetic tree of strains Red96^T, Red100^T, Red88^T and representatives in the order *Desulfuromonadales* based on 16S rRNA sequence divergence. The tree was inferred by maximum-likelihood algorithm using MEGA 7.0 with K2+G+I model. The background colours represent different bacterial genera. Bootstrap values (expressed as percentages of 1,000 replications) are shown at branching nodes with black or grey dots. Bar, 0.1 substitutions per nucleotide position.

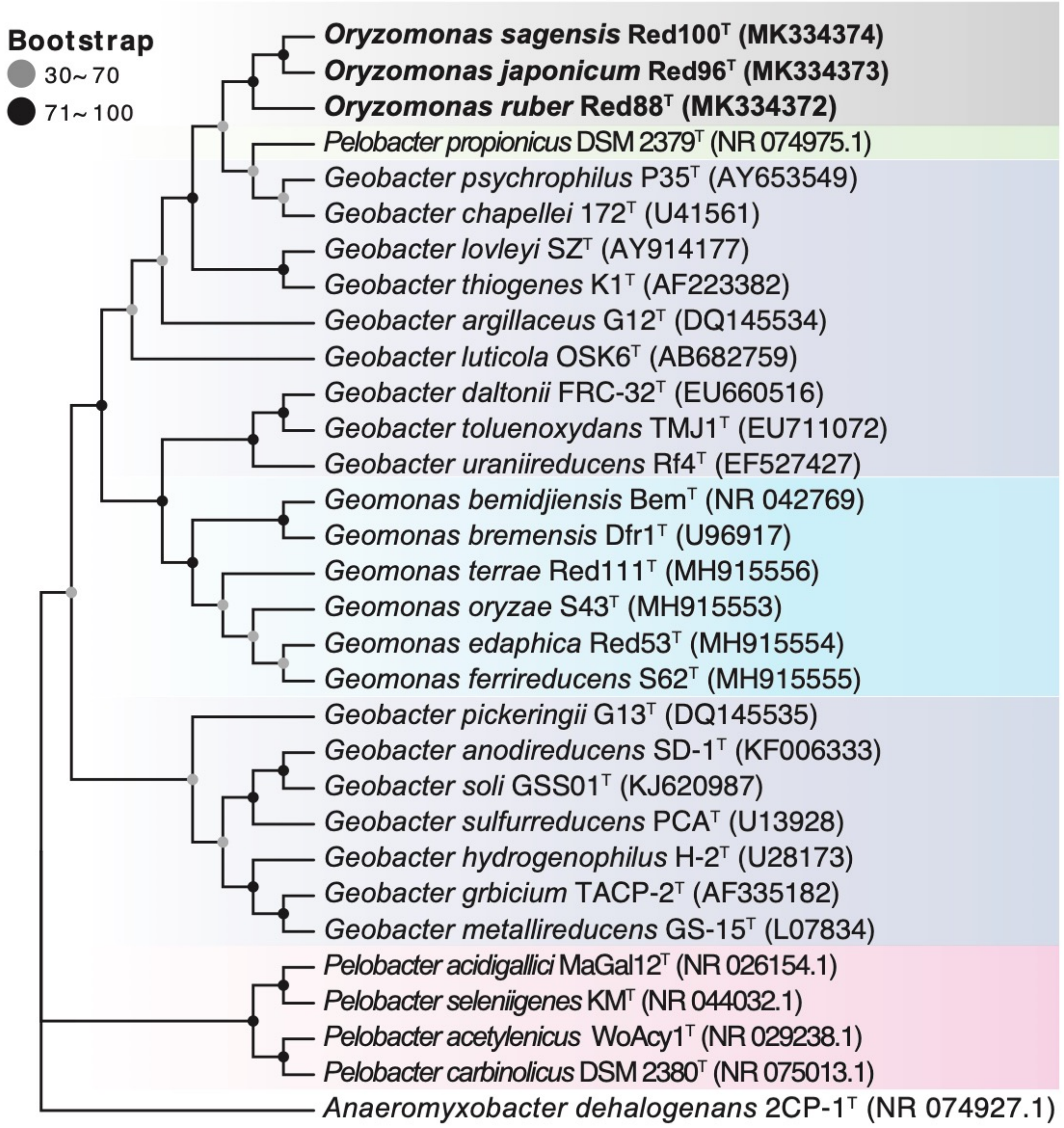

Figure S4. Phylogenetic tree of strains Red96^{T}, Red100^{T}, Red88^{T} and representatives in the order *Desulfuromonadales* based on 16S rRNA sequence divergence. The tree was inferred by maximum-parsimony algorithm using MEGA 7.0. The background colours represent different bacterial genera. Bootstrap values (expressed as percentages of 1,000 replications) are shown at branching nodes with black or grey dots.

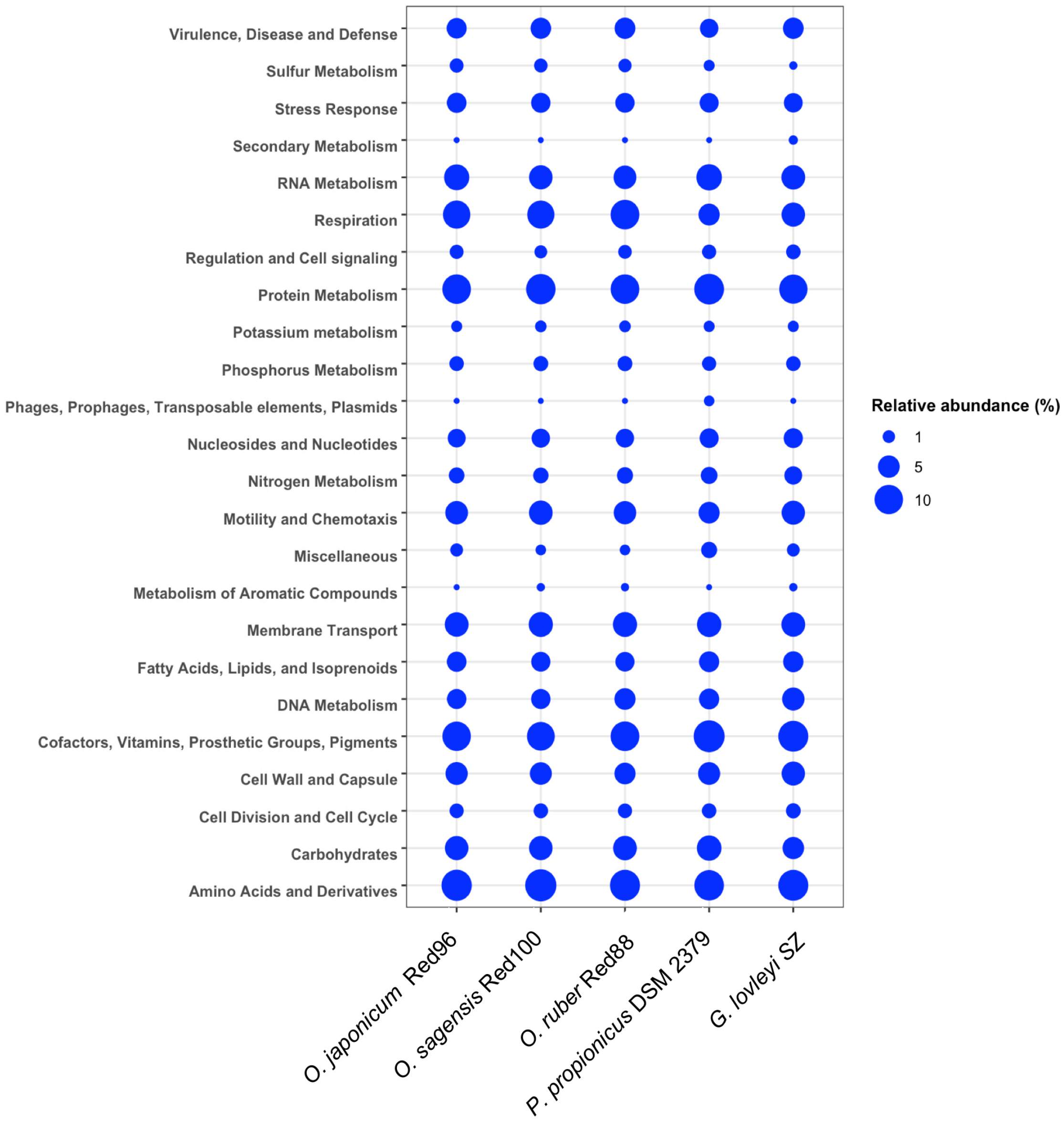

Figure S5. The gene proportion of different functional categories for the three isolated strains: *Oryzomonas japonicum* Red96^T, *Oryzomonas sagensis* Red100^T, *Oryzomonas ruber* Red88^T and their two neighbors: *Pelobacter propionicus* DSM 2379^T and *Geobacter lovleyi* SZ^T. The genes derived from the whole genomes are annotated and classified by RAST (http://rast.nmpdr.org/rast.cgi).

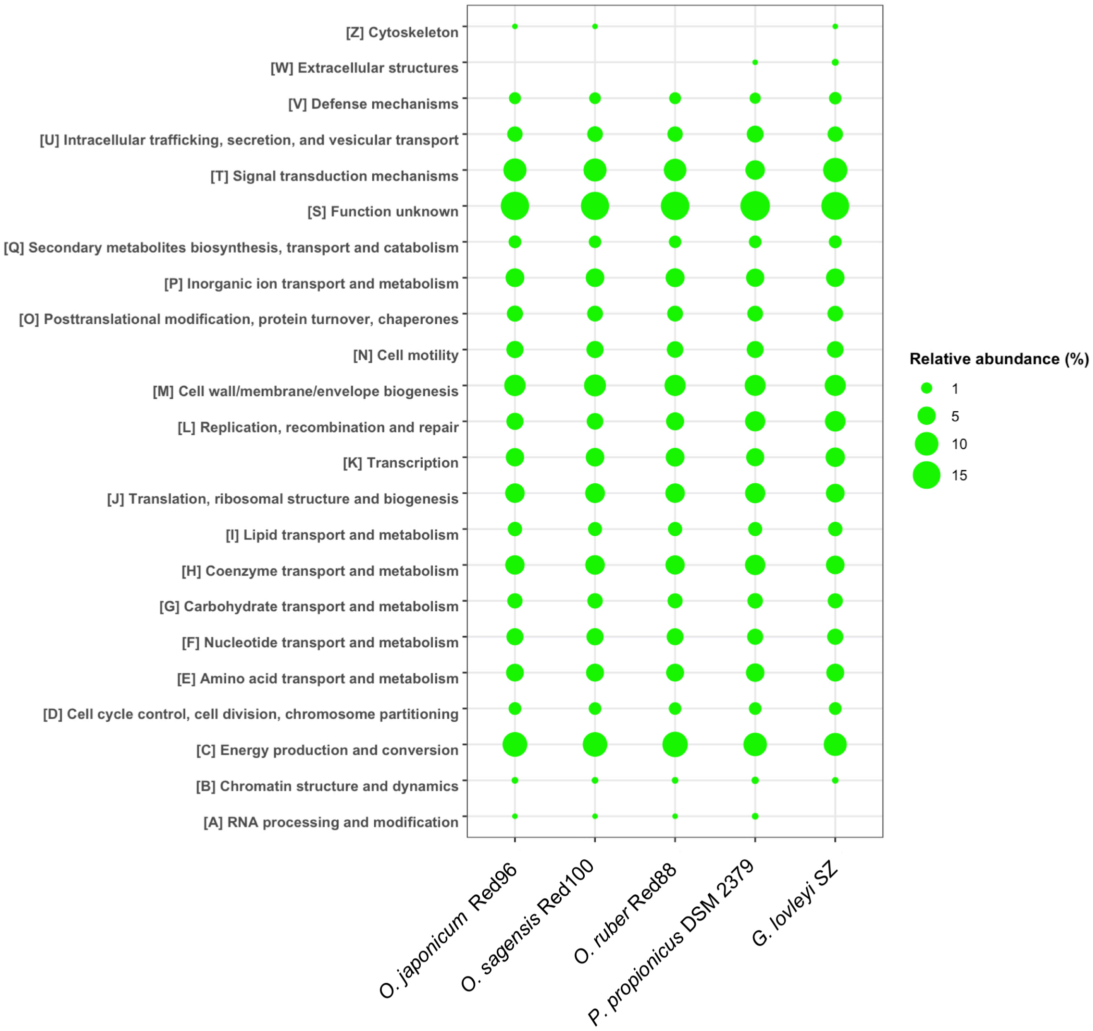

Figure S6. The gene proportion of different functional categories for the three isolated strains: *Oryzomonas japonicum* Red96^T, *Oryzomonas sagensis* Red100^T, *Oryzomonas ruber* Red88^T and their two neighbors: *Pelobacter propionicus* DSM 2379^T and *Geobacter lovleyi* SZ^T. The genes derived from the whole genomes are annotated and classified based on eggNOG database (http://eggnogdb.embl.de/#/app/home).

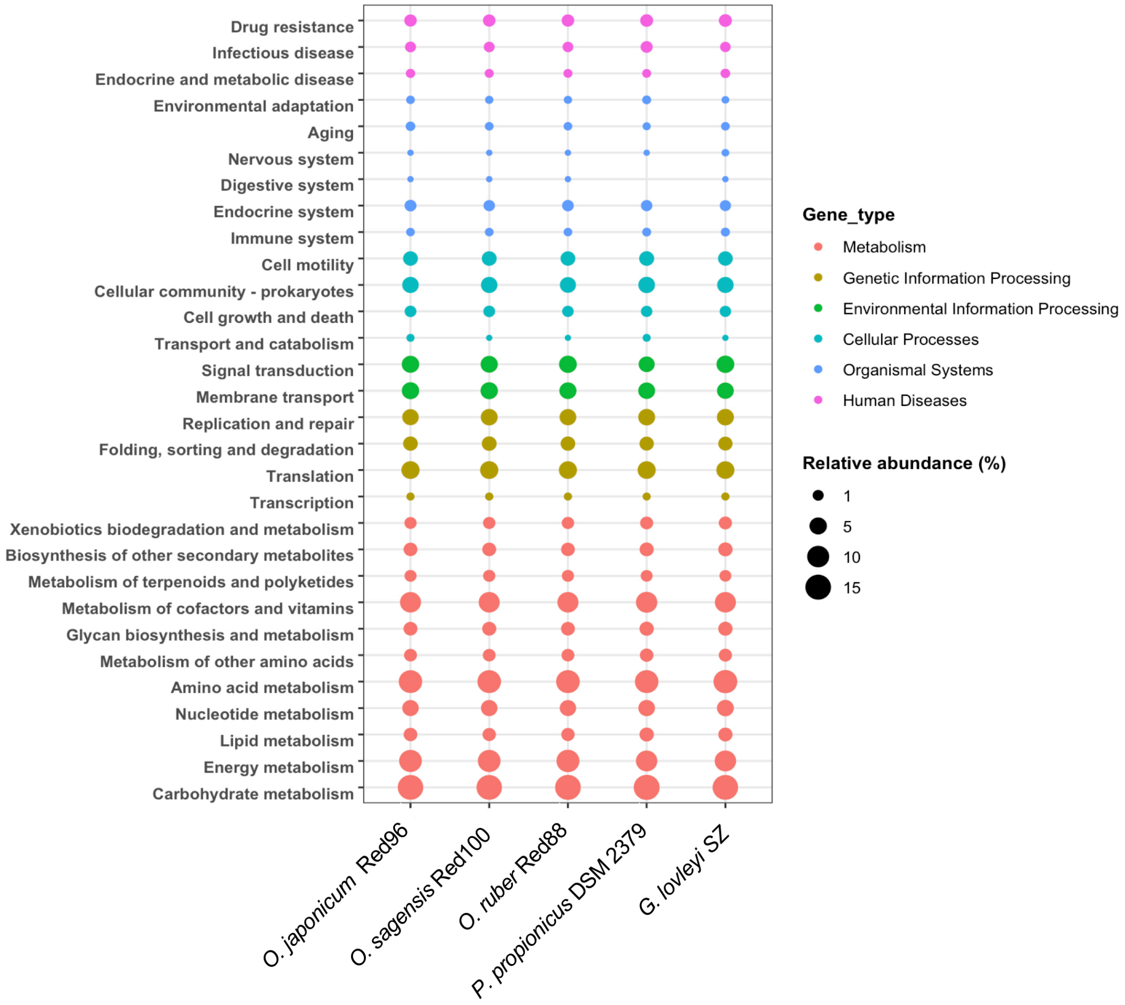

Figure S7. The gene proportion of different functional categories for the three isolated strains: *Oryzomonas japonicum* Red96^T, *Oryzomonas sagensis* Red100^T, *Oryzomonas ruber* Red88^T and their two neighbors: *Pelobacter propionicus* DSM 2379^T and *Geobacter lovleyi* SZ^T. The genes derived from the whole genomes are annotated and classified by KEGG (https://www.kegg.jp/).

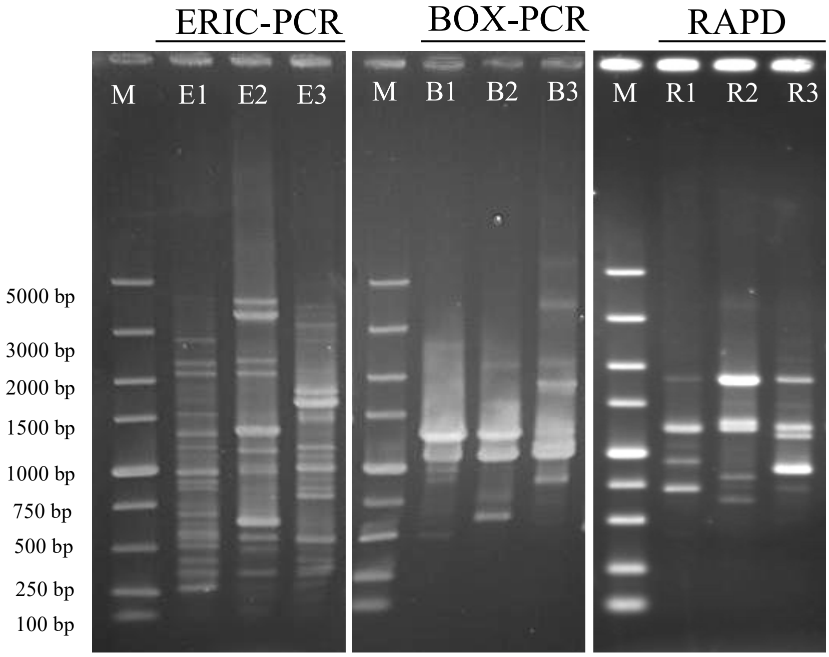

Figure S8. Agarose gel-electrophoresis with rep-PCR and RAPD patterns of strains Red88^T, Red96^T and Red100^T. ERIC-PCR indicates rep-PCR with ERIC primers and BOX-PCR indicates rep-PCR with BOX primer. E1, B1 and R1: *Oryzomonas ruber* Red88^T; E2, B2 and R2: *Oryzomonas japonicum* Red96^T; E3, B3 and R3: *Oryzomonas sagensis* Red100^T; M: DNA marker.
